# Progenitor diversity during formation of the mammalian neocortex

**DOI:** 10.3389/fnins.2026.1834625

**Published:** 2026-05-26

**Authors:** Zeynep Atak, Tarik F. Haydar

**Affiliations:** Department of Anatomy and Neurobiology, Boston University School of Medicine, Boston, MA, United States

**Keywords:** autism spectrum disorder (ASD), Down syndrome, neocortex, neurodevelopment, neurogenesis, neuronal lineage, precursor

## Abstract

Mammalian neocortical development follows a precise spatiotemporal sequence to generate the organized structure responsible for higher-order cognition and behavior. Increasing evidence suggests that diversification of neural stem and progenitor cells during prenatal development is a key step in the emergence of the intricate circuitry and functional architecture of the cerebral cortex. This review discusses novel findings with an emphasis on mechanisms and consequences of cell lineage variation during normal and altered brain development, including focus on neurodevelopmental disorders such as autism spectrum disorders and Down syndrome.

## Origins of neocortical diversity

Neocortical circuitry constitutes one of the most complex biological systems due to the elaborate connections between different cell types, brain areas, and body systems. How this functional architecture is planned and executed from early periods of brain development, and how it can be so successfully replicated in millions of individuals, have not yet been explained. Multiple intrinsic mechanisms and extrinsic cues are needed to properly pattern the forebrain; recent data indicate that different contemporaneous groups of precursor cells in the fetal brain generate neurons with distinct characteristics, indicating that precursor diversity plays an important role in generating neuronal diversity as brain areas develop. How these different precursor cells impact neocortical circuit complexity and function is the subject of this review.

The mature neocortex is composed of six layers and is postulated to have first appeared in the early mammals (Kaas, 2011), with perhaps an appearance of a neocortex-like structure in non-mammalian synapsids of the late Permian (Laass and Kaestner, 2017; Goffinet, 2017). A laminar neocortical structure is observed in all mammals and follows a stereotypical organization; however, there are large differences in size between species and the neocortex can either develop convolutions (gyrencephaly) or remain smooth (lissencephaly) (Sun and Hevner, 2014). Precursor cell activity has been theorized to underpin both overall cortical growth and its degree of gyrification. Remarkably, the diversity of neuron and glial cells in the neocortex arises from a germinal zone containing a relatively simple sheet of neuroepithelial progenitor cells (NEPs) (Martinez-Cerdeno and Noctor, 2018; Morest and Silver, 2003). These NEPs give rise to a large number of precursor types, and this rapid molecular and morphological diversification of precursors within the germinal zone is now recognized to be a critical feature of proper brain development.

## Cortical precursor variety

NEPs initially undergo exponential growth via symmetrical divisions as they form the first layer of the developing telencephalon, the ventricular zone (VZ) (Sidman et al., 1959). The duration of this exponential growth phase is different between mammalian species and is thought to directly correlate with overall brain size (Rakic, 1995; Caviness et al., 1995). Before neurogenesis begins, NEPs transform into apical radial glial cells (aRGCs), the resident stem cells of the prenatal neocortex. Over the course of neurogenesis, aRGCs undergo asymmetrical divisions that enable their self-renewal while producing postmitotic neuron daughter cells that migrate to the growing cortical plate. At the same time, aRGCs also divide asymmetrically to generate at least three different morphological classes of intermediate neurogenic precursors which continue to produce neurons from the VZ and the subjacent subventricular zone (SVZ). These intermediate progenitors include the apical intermediate precursor cells (aIPC) and truncated radial glia (tRGC) which are short bipolar VZ cell types whose processes do not extend across the neocortical wall, the multipolar basal intermediate precursor cells (bIPC) that reside in the SVZ, and the basal radial glia (bRGC) progenitors that inhabit the outer SVZ and which can adopt multiple morphologies. Importantly, these intermediate precursors and their parent aRGCs all cohabit the germinal zones in the mammalian brain and divide throughout the neurogenesis period to amplify the postmitotic neuron output during fetal neurogenesis.

**Figure 1:**
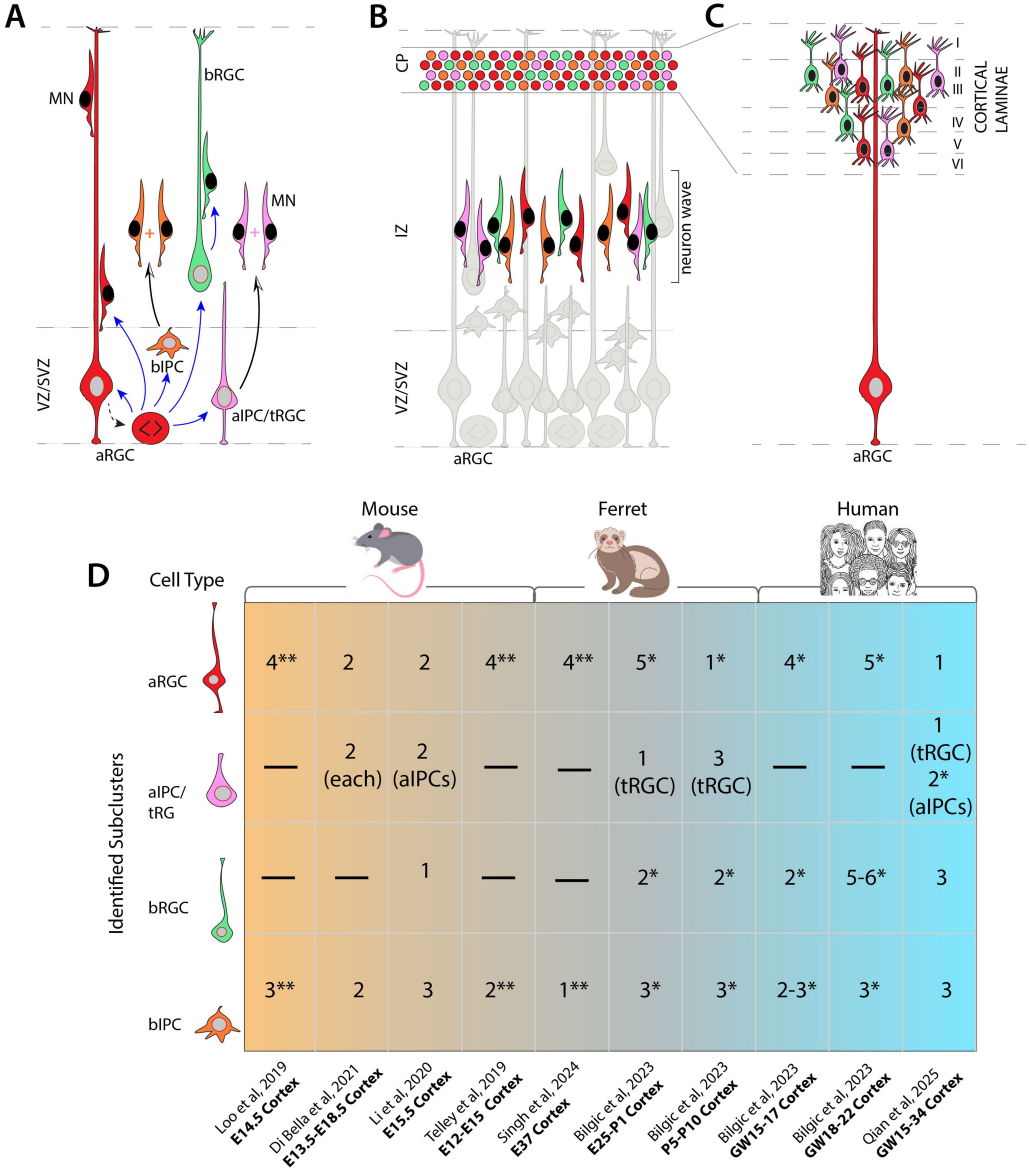
Neocortical progenitor heterogeneity across mammals. **(A)** Schematic illustrating the different morphological groups of neural precursors and the neuron daughter cells they produce. Note that aRGCs and bRGCs predominantly divide asymmetrically to self-renew while producing neurons and IPCs whereas bIPCs and aIPCs/tRGCs divide symmetrically. **(B)** Illustration of a wave of migrating neurons just produced from the germinal zones, depicting their lineage of descent by parent progenitor color denoted in **(A)**. Thus, each new layer of the neocortex is formed by neurons from diverse parentage. **(C)** Diagram showing the radial unit produced by one parent aRGC. The neurons directly and indirectly related to one aRGC mother cell populate all cortical layers. **(D)** Comparative summary of transcriptomic studies in rodent, ferret, and human cerebral cortex, showing the number of transcriptionally defined clusters assigned to each major morphologically defined progenitor class. In some studies, these classes were further subdivided into finer groups (indicated where applicable), whereas in others no additional subdivision was performed (asterisks). Therefore, reported cluster numbers reflect differing levels of analytical resolution rather than equivalent biological granularity. Because datasets vary in species, developmental stage, sampled region, and analytical approach, cluster counts are not directly comparable across studies. This panel is intended to illustrate overall progenitor diversity across species, not one-to-one correspondence between datasets. VZ, ventricular zone; SVZ, subventricular zone; IZ, intermediate zone; CP, cortical plate; MN, migrating neuron; aRGC, apical radial glial cell; bRGC, basal radial glial cell; bIPC, basal intermediate progenitor cell; aIPC, apical intermediate progenitor cell; tRGC, truncated radial glial cell. *Major cell types inferred from key markers, not divided further. May reflect transitional states. ** Study did not further divide apical and basal progenitors.

Thus, the basic modular unit of excitatory neuron production in the neocortex appears to consist of 4 major morphologically different cell types (aRGCs, aIPCs/tRGCs, bIPCs, and bRGCs) that together produce the neurons for each lamina over the course of prenatal neurogenesis (Figures 1A,B). This proliferative module, and the overlying cortical neurons it produces, together form a “radial unit” (Figure 1C) (Rakic, 1988). If the parameters controlling this module are fixed, then repeating the module throughout the VZ should generate an expanded mantle with identical laminar features (Rakic and Caviness, 1995). Indeed, most cortical areas are isolaminar, indicating that the mechanisms controlling the growth of their derivative radial units were constant across a given area. However, the multicellular nature of the module also endows it with many adjustable elements (e.g., different asymmetrical divisions to favor/disfavor specific daughter cell types, or by altering the neuronal output of individual cell types through signaling or gene expression changes). Therefore, adjusting the module may lead to changes to the resultant radial unit, and sharp laminar distinctions between neighboring cortical areas, such as between BA17 and BA18, indicate that the underlying proliferative modules in these areas may be different (Kim et al., 2023).

This potential plasticity of module function can lead to mild or substantial laminar consequences. Temporary changes (or “blips”) in the developmental plan of each module can also occur, some of which can lead to small but consequential changes in neuronal allocation and circuit function. Alterations to the proliferative module during prenatal development have now been implicated in Down syndrome and autism, often without conspicuous anatomical changes in the overlying cortical plate (Atak et al., 2025; Pan et al., 2019). Future, rigorous testing of cortical module composition is required to fully understand how small changes in development can lead to lasting effects on function. This effort requires new tools for identifying individual NPC subtypes and for measuring their contribution to the overlying circuitry in question. Novel single-cell molecular, physiological, and anatomical methods are beginning to dissect the origins of these lineage differences and to show how this developmentally regulated burst of cell variety is used to create the discrete circuits that underlie function.

### Lineage specification mechanisms

Uncovering the diversity of the brain’s progenitor cells is a major recent advance in our understanding of mammalian brain development. The demonstration that these different progenitor groups make unique contributions to formation of the cerebral cortex could fundamentally impact clinical approaches to brain alterations during development and/or aging. Initial evidence of molecular heterogeneity in neuronal progenitors of the dorsal telencephalon came from the observation of glial fibrillary acidic protein (GFAP) expression in some (but not all) of the VZ precursors in the fetal rhesus monkey (Levitt et al., 1983). Differential gene expression among neural precursor cell types has since become well-established. For example, both aRGCs and bRGCs express the neural stem cell markers Pax6 and Sry-box2 (Sox2), which are critical to RGC differentiation and self-renewal, respectively (Gotz et al., 1998; Subramanian et al., 2017). However, in contrast to the majority of RGCs, bIPCs express the transcription factor T-box brain protein 2 (Tbr2/Eomes). Basal IPCs differ from aRGCs by location, morphology, division behavior and gene expression. Although Tbr2 protein expression appears to be specific to IPCs, it was recently found that *Tbr2* transcripts can be produced by aRGCs but are translated into protein only by their IPC progeny following cell division. This phenomenon, called transcriptional priming, appears to be conserved between rodents and humans (Li et al., 2020). Gene expression differences between species are also evident, as many bRGCs in the mouse also express both *Sox2* and *Tbr2*, while a majority of human bRGCs do not (Florio et al., 2015; Englund et al., 2005). Interestingly, apical intermediate progenitors (aIPCs) undergo symmetric neuron-generating cell divisions like bIPCs, but they do not express Tbr2 protein (Gal et al., 2006). Instead, they express Pax6 and undergo interkinetic nuclear migration (INM) and mitosis in the VZ like aRGCs. This indicates that all intermediate neural precursors may not express Tbr2 in all species and that a “symmetric-out” division character may better combine them into a functional group.

Emerging single cell transcriptomic evidence suggests that these IPC classes can be further divided into subclasses based on divergent gene expression profiles between different “clusters” of bIPCs (Figure 1C) (Li et al., 2020; Pfeiffer et al., 2016; Pilz et al., 2013; Loo et al., 2019; Di Bella et al., 2021; Telley et al., 2019; Singh et al., 2024; Bilgic et al., 2023; Qian et al., 2025). For example, in some transcriptome studies where apical or basal precursor groups have been bioinformatically re-clustered to discover differences within their morphological cell type, multiple contemporaneous but separate populations of bIPCs and aRGCs have been identified (Figure 1D), However, assigning functional precursor identity to these transcriptional subtypes is not straightforward, as transcriptomic data provides only a static snapshot of mRNAs in a cell population and is therefore upstream of many key phenotypic determinants of cell function (e.g., proteome, post-translational modifications, RNA modifications) which are not captured by mRNA sequencing. In addition, it remains unclear whether transcript variation represents temporary changes in cell state versus genuine, lasting variance in cell identity or fate. This lack of clarity on the true level of progenitor variety needs resolution, especially given the evidence that proportional changes to progenitor subclasses can induce substantial alterations to neocortical structure and function.

Cortical neuron specification is constrained by tight rules of layer production during development, ensuring a high degree of cellular diversification within a consistent and temporally regulated laminar architecture. Initial studies describing neocortical development provided important but limited understanding of the mechanisms controlling cortical progenitors, as at the time it was thought that progenitors within the neuroepithelium mostly behave uniformly (Takahashi et al., 2002; Takahashi et al., 1999). This was exemplified by the Waddington model of neurogenesis, whereby the developmental trajectory of an aRGC stem cell in the VZ is determined by the “epigenetic landscape” which functions to direct cell fate from a fixed ground state of open and closed genomic loci (Goldberg et al., 2007). However, subsequent lineage tracing and heterochronic cell transplant experiments demonstrated a remarkable degree of fate plasticity of cortical progenitors (Walsh and Cepko, 1993; McConnell, 1988), indicating that not all progenitors have equivalent ground states. For instance, when early-born cortical progenitors are transplanted to a more mature cortex, they can generate neurons that populate (later-born) superficial cortical layers (McConnell and Kaznowski, 1991). In contrast, late-born progenitors transplanted to more immature cortex cannot generate neurons with properties relevant for the deep, earlier-born layers, indicating that progenitors undergo progressive restriction in fate plasticity over the course of neurogenesis (Frantz and McConnell, 1996; Desai and McConnell, 2000). These observations were later confirmed and extended with isochronic fate mapping of aRGC pools with the FlashTag pulse labeling technique, showing both a temporal progression as well as a dynamic succession of transcriptional ground states from which lineage restriction can occur, all of which contrast the prior Waddington model (Telley et al., 2019). The new fate specification paradigm also introduces the potential for stochastic cell fate decisions that can modify the overall output in terms of numbers of neurons with specific functional attributes (Johnston and Desplan, 2010). It is important to also recognize that the system can employ compensatory plasticity, whereby intrinsic and extrinsic mechanisms may “correct” for adverse disruptions to the neurogenesis process either while cells are still within the germinal zones or during and after neuronal migration (Johnston and Desplan, 2010). Cortical formation is affected by external cues as well, for example by reacting to components of the extracellular matrix or differential sensory inputs [e.g., neocortical layer 4 pyramidal neurons require signaling from thalamic afferents for their maturation (Gerstmann et al., 2015)], or to interactions with glial cells and inhibitory neurons (Telley and Jabaudon, 2018). Thus, unmixing the intrinsic versus extrinsic drivers of a neuron’s character is crucial but also very difficult since intrinsic and extrinsic cues often overlap spatially and temporally.

Advances in live imaging and super-resolution microscopy allow tracking of cell fate following different modes of mitotic cleavage and may thus address these gaps in understanding (Godin et al., 2014; Breedijk et al., 2020). A recently developed imaging methodology, termed temporally multiplexed imaging, uses the variable kinetics of different photoswitchable fluorescent proteins to enable multiplexed live imaging of different intracellular targets (Qian et al., 2023). Using these new techniques, one possible approach would be to genetically label and track the mitotic behavior of different progenitor classes over time to obtain high-resolution dynamic information about their roles during neocortical development.

### Progenitor heterogeneity: cortical expansion through an evolutionary lens

Multiple lines of evidence indicate that higher mammals utilize different cortical progenitor cell classes in unique manners to produce increases in surface area and computational power. Focusing on the primate order, the human neocortex is approximately three times the size of the chimpanzee cortex and contains twice as many neurons (Collins et al., 2016; Balzeau et al., 2012). Among the key reasons for the staggering expansion of the human neocortex (estimated to be a 1,000-fold increase compared to the mouse) is the overall increase in the number of progenitor cells in the VZ and the substantial increase in indirect neurogenesis via basal progenitors (e.g., higher numbers of bRGCs within the oSVZ) (Reillo et al., 2011). This neocortical expansion through increased numbers of individual progenitor classes might enhance neural information processing capacity, especially if it produces additional neurons with distinct membrane and electrophysiological capacities.

General characteristics of cortical neuron allocation were first discovered in experiments on the developing non-human primate brain, in which the neocortical laminar structure was shown to develop in an “inside-out” fashion (Rakic, 1975a; Rakic, 1971). In particular, earlier-born neurons form the deeper cortical layers and later-born neurons migrate past these older neurons to establish superficial layers (Rakic, 1975b). However, in contrast to the exceptionally sharp temporal specification of cortical layers in the primate brain, a recent study in the mouse has shown that contemporaneously born neurons end up occupying different laminae in the neocortex in a manner more consistent with their future axonal projection subtype rather than their day of birth (Huilgol et al., 2025). These new results indicate that intrinsic potential may be the arbiter of a neuron’s position rather than day of birth, at least perhaps for rodents. This apparent discrepancy will need to be addressed by future studies aimed at deciphering whether and how presumptive cortical lamination and connectivity patterns are species-specific or encoded differently in distinct progenitor lineages.

Diverse progenitor characteristics may also be a key determining factor governing the formation of a lissencephalic (smooth) or gyrencephalic (convoluted) cortex. Indeed, progenitor proliferation and cortical expansion are thought to be directly correlated with the magnitude of cortical gyrification. For instance, neocortical development in gyrencephalic non-human primates occurs over a period of prolonged neurogenesis, which is augmented by lengthening of the gestational period to allow for the maximum number of divisions. Several hypotheses have been proposed to explain how differential neurogenesis might underlie cortical gyrification, including the radial expansion (Stewart et al., 1975), differential tangential expansion (Ronan et al., 2014) and axonal tension (Van Essen, 1997; Holland et al., 2015) hypotheses. Radial expansion posits that selective overproduction of superficial pyramidal neurons compared to deep layer cells can result in localized widening of the superficial layers and the emergence of a gyrus. Tangential/lateral expansion refers to neocortical regions undergoing different rates of tangential spreading, thus eventually leading to formation of gyri and sulci. Under the axonal tension hypothesis, axonal dendrites and their differential extension drive regional neocortical folding. It postulates that localized increases in corticocortical axonal fiber extension can create focal regions of mechanical tension, inducing neocortical surface folding and gyrification. These models inherently rely on differential neurogenesis to establish region-specific patterns of tissue expansion or neuronal connectivity which in turn generate the variable mechanical forces necessary for neocortical folding. Ultimately, each gyrification model implicates distinct neurogenic pathways driven by specific progenitor classes, whose spatial and temporal dynamics shape the patterns of cortical expansion and folding characteristic of the developing neocortex.

Work with gyrencephalic species has begun to uncover progenitor lineage-specific contributions to cortical folding and nascent gyrus formation. For example, in the domesticated ferret (*Mustela putorius furo*), in which all convolutions appear after birth, the oSVZ is highly expanded compared to lissencephalic rodent brains (Gertz et al., 2014). Homeodomain-Only Protein Homeobox (HOPX) expressing bRGCs have been shown to be critical for correct cortical folding in the ferret (Gertz et al., 2014; Matsumoto et al., 2020), and evidence suggests that distinct and specific transcriptional signatures can be found in the oSVZ underlying future gyrus and sulcus sites (de Juan Romero et al., 2015). In addition to HOPX, elevations in Tbr2 expression in prospective gyri was linked to a larger bIPC and bRGC pool. Correspondingly, suppression of Tbr2 reduced these progenitor populations and disrupted upper-layer expansion, preventing normal gyrus and sulcus formation (Toda et al., 2016). These findings argue for the prospective specification of gyrification sites by germinal zone cells underlying the site(s) of convolution. Further evidence for the importance of basal progenitor lineages in cortical gyrification comes from transcriptomic studies of human and mouse progenitor subpopulations, in which the human gene *ARHGAP11B* gene was found to promote IPC identity when expressed in radial glia (Florio et al., 2015). Once *ARHGAP11B* was expressed in mouse (Florio et al., 2015), ferret (Kalebic et al., 2018) and marmoset (Heide et al., 2020) developing cortex, it elicited increased bRGC self-renewal, enhanced gyrification, and neocortical enlargement in all model species, strongly arguing for its causative role in cortical convolutions.

Other studies have also linked bIPC and bRGC activity to gyrification. For example, their enhanced proliferation due to constitutively active signaling through the Sonic Hedgehog (SHH) pathway is sufficient to induce ectopic gyrus-like outgrowths in the mouse cingulate cortex (Wang et al., 2016). Interestingly, aRGC proliferation was found to be unperturbed in these mutants, strongly suggesting that basal progenitor expansion is the key driver behind the localized cortical expansion observed in the mouse. Another mouse study found that manipulating levels of the *Trnp1* gene in cortical progenitors was sufficient to bias their behavior between self-renewal/tangential expansion and bIPC differentiation/radial expansion, providing a basis for how gyrification may be initiated (Stahl et al., 2013). However, while these outgrowths in mouse neocortex might indicate how the foundations of presumptive cortical gyri form, full and proper gyrification likely depends on other mechanisms absent from the lissencephalic mouse brain. In comparative studies, the overall temporal sequence of aRGC to bIPC differentiation is well conserved between humans, non-human primates, and rodent models of corticogenesis. However, the process of bRGC generation and differentiation differs markedly between macaques and rodents at the transcriptomic level, likely as a result of fewer bRGCs being specified in the rodent cortex (Xu et al., 2024; Micali et al., 2023). Diversification of this particular precursor type is extended in both humans and macaques, pointing to a prominent role for bRGCs in organizing the convoluted cortex.

While studies in both ferret and rodent models have provided valuable insights into how specific signaling pathways influence progenitor behavior and the consequent cortical expansion, these models also highlight species-specific constraints that limit our ability to fully capture the evolutionary complexity of neocortical gyrification. Conclusive studies will require the access and study of gyrencephalic brains in a species that affords the possibility of molecular, imaging and longitudinal *in vivo* studies. In addition, comparative approaches making use of *in vitro* models such as induced pluripotent stem cells (iPSCs) and ex vivo preparations (e.g., organotypic slice culture) from gyrencephalic model species will enhance the rigor and translational relevance of any findings of progenitor heterogeneity.

While cortical expansion and gyrification are key developmental processes that distinguish one species from another, comparative neuroanatomical work has hinted at another precursor population that may differ among mammalian species. In addition to being the site of origin of most supragranular/superficial layer pyramidal neurons, the oSVZ in gyrified brains may also be the termination point of cell processes of truncated radial glial cells (tRGC), which do not contact the pial surface and are discontinuous with the fibers of the oSVZ-based bRGCs (Nowakowski et al., 2016). In the ferret, these truncated progenitors were shown to eventually differentiate into astroglial and ependymal cells lining the ventricular space (Bilgic et al., 2023). The tRGCs found in gyrified brains morphologically resemble the apical intermediate precursors (aIPCs) found in mouse (Gal et al., 2006), and it remains unclear whether there are functional, transcriptomic, or lineage similarities between these two cell types. One possibility is that both tRGCs and aIPCs form upon generation of a bRGC from an aRGC – they are perhaps the daughter cell left behind with a foot process at the ventricle while the other (bRGC) daughter cell inherits the ascending process and moves into the oSVZ. Another possibility is that tRGCs are aRGCs that have either retracted their pia-contacting foot process or suffered a traumatic break in this long process by external means. Regardless of how these short precursor types are formed, whether tRGCs and aIPCs share molecular identities and functional roles during brain development has not yet been established. Resolving the origin, roles, and evolutionary conservation of tRGCs is an important future avenue of research, as it will help uncover whether and how they structurally contribute to neocortical development.

### Progenitor heterogeneity: effects on cortical circuitry

Another specific role for progenitor diversity may be to propagate the various intrinsic states and axonal targeting patterns that are needed for the mature cortex. In particular, birth-dating, clonal tracing, and genetic fate mapping studies have shown that neurons found in the same cortical layer, yet originating from different progenitor lineages, have distinct electrophysiological and morphological properties (Tyler and Haydar, 2013; Tyler et al., 2015; Guillamon-Vivancos et al., 2019; Ellender et al., 2019). For example, pyramidal neurons derived from Tbr2-expressing IPC lineages (via indirect neurogenesis) exhibit higher input resistance and respond to depolarizing current with higher frequency action potentials compared to neurons directly produced from aRGCs (Tyler et al., 2015). In addition, Tbr2 lineage neurons have reduced dendrite complexity compared to neurons directly produced from aRGCs, particularly in the apical dendritic arbor. While each cortical layer may be composed of neurons originating from different lineages, the clonal progeny of the same aRGC can disperse across multiple layers (Figure 1C) (Walsh and Cepko, 1993; Tan et al., 1995), providing evidence of an evolutionary strategy to generate diversity both horizontally and vertically in the neocortical wall. Due to the specific functional characteristics and diverse settling behaviors of the neurons in the fetal neocortex, the admixture of different progenitor lineages may be the engine that drives the diversity of structural and functional connectivity in the neocortex. The larger, more complex human embryonic neocortex must therefore result either from enhanced output from the same number of precursor types found in all mammals, or the human brain may harbor a greater number of distinct progenitor classes compared to species with small, lissencephalic brains. Notwithstanding the aforementioned caution of interpreting bioinformatically-generated clusters of cells as individual types versus cell states, the evidence thus far supports both mechanisms, given the greater overall numbers of progenitor subtypes/states bioinformatically identified in the human embryonic neocortex compared to the mouse, with the highest estimates pointing to 24 collective radial glia clusters in the human neocortex across post-conception weeks 3–12 (Loo et al., 2019; Zeng et al., 2023). The increased progenitor diversity in human brain likely points to more elaborate cell type-specific regulation in the germinal zones and raises the possibility that other gyrencephalic species may also have increased types or states of precursor cells (Pollen et al., 2019; Kanton et al., 2019; Benito-Kwiecinski et al., 2021).

More recent efforts have begun confirming the laminar contributions of different cortical progenitor lineages. For example, an intersectional labeling strategy using multiple *Cre-lox* and *Flp/FRT* transgenic mouse lines fate mapped pyramidal neurons born from the direct (via aRGC) and indirect (via IPC) neurogenesis pathways (Huilgol et al., 2023). The findings show that aRGCs contributed to all major pyramidal neuron classes but most heavily produced neurons for deep layer (layers V/VI). However, the indirect pathway contributed to the vast majority of superficial layer (cortical layers II/III) pyramidal cells. Thus, the direct and indirect neurogenesis pathways contribute differently to the various projection subtypes present among pyramidal neurons (Huilgol et al., 2023). These subtypes include locally-projecting intratelencephalic (IT) pyramidal cells (predominantly originating from indirect neurogenesis), subcortically projecting pyramidal tract (PT) neurons (mixed origin) and corticothalamic (CT) neurons exclusively targeting the thalamus (predominantly direct neurogenesis). Multiple lines of evidence now show that different progenitor lineages produce neurons with distinct membrane properties and intra−/extra-cortical connectivity patterns. This potential to generate variations in excitability and connectivity provides the framework supporting complex circuit properties.

## Lineage specificity in neurodevelopmental disorders

The correlation between cortical neuron lineage and fate assignment discussed above requires tight control over multiple progenitor populations, including their physical coordinates in space, control of the timing of the birth and migration of their progeny, and the passage of molecular characteristics to their daughter cells. Modifications to the developmental sequence, even within a single progenitor pool, can result in significant changes to cortical formation. In line with these observations, humans with homozygous mutations in *TBR2*, which depletes their bIPC pool, suffer from microcephaly and white matter deficits (Li et al., 2018; Castren, 2016; Aziz et al., 2018; Chakrabarti et al., 2007; Guidi et al., 2011). Divergent prenatal development may also be a hallmark of several different neurodevelopmental disorders, ranging from chromosomal abnormalities such as trisomy 21/Down syndrome (DS) to autism spectrum disorders (ASDs) (Mato-Blanco et al., 2025). It is increasingly clear that developmental alterations to the proliferative module depicted in Figure 1A may directly influence circuit-level changes in affected individuals throughout the lifespan. For example, tissues obtained from fetuses with DS (17–21 weeks gestation) exhibit reduced proliferation of NPCs in the hippocampal dentate gyrus and germinal zones of the lateral and third ventricles prior to the onset of cerebral volumetric changes (Contestabile et al., 2007; Stagni et al., 2020). This reduction in neurogenesis likely causes a depletion in the NPC pool, and fewer SOX2 + progenitors were reported in the outer subventricular zone (oSVZ) of the developing DS cerebral cortex during mid-gestation (18–24 weeks) (Baburamani et al., 2020). These changes in NPC proliferation and survival are correlated with hypocellularity across multiple brain structures throughout development (Baburamani et al., 2020), including the significant cell loss observed in the forebrain at 19 weeks gestation (Larsen et al., 2008). Reports have also identified reductions in cellular density in other regions such as the superior temporal neocortex, but only after 23 weeks gestation, suggesting regional and temporal specific drivers of the neurogenic deficits in DS individuals (Schmidt-Sidor et al., 1990; Golden and Hyman, 1994). Altogether, these data indicate that proliferative modules across the cortical sheet may be altered in different regions and at different times.

Animal model studies have demonstrated cortical progenitors to be sensitive to altered transcriptional mechanisms and gene dosages associated with neurodevelopmental disorders such as DS. For instance, the extra chromosome 21 in DS influences fetal brain development by prolonging cell cycle duration and increasing apoptosis in neocortical NPCs (Contestabile et al., 2007; Guidi et al., 2008; Guidi et al., 2018; Stagni et al., 2018), leading to microcephaly and cortical dyslamination (Golden and Hyman, 1994). Importantly, as seen in the Ts65Dn mouse, this process also includes a temporary increase in bIPC proliferation which may represent a compensatory response to the underproduction of neurons (Chakrabarti et al., 2007). Animal models reinforce these findings: the Ts65Dn mouse exhibits prolonged aRGC cell cycles and reduced progenitor output (Aziz et al., 2018; Chakrabarti et al., 2007), while the humanized TcMAC21 model shows transient mid-gestation deficits specifically in bIPCs, leading to long-term impairments in cortical layering and activity (Atak et al., 2025). These results uncover the specific sensitivity of distinct progenitor classes to trisomy 21 and suggest that even temporally limited disruptions during critical windows of neurogenesis can produce lasting changes in neocortical architecture and function.

The risk of changes to brain precursor cells also appears to apply in autism spectrum disorders (ASD). Multiple genetic and environmental animal ASD models have revealed disruptions in specific progenitor populations, often involving aberrant signaling pathways such as ERK/MAPK and mTOR. In the 16p11.2 model, for example, overactive MAPK signaling enhances progenitor proliferation but accelerates cell cycle exit, depleting the progenitor pool and leading to an imbalance in projection neuron subtypes. Dysregulated mTOR signaling specifically alters bRGC morphology and division without affecting proliferation, underscoring a potentially unique vulnerability of oSVZ progenitors (Andrews et al., 2020). Environmental models, including maternal immune activation and valproic acid exposure, similarly impact the generation or function of bRGCs and superficial-layer neurons (Zang et al., 2022; Dorsey et al., 2024).

Taken together, these findings suggest that distinct but converging disruptions in progenitor subtypes, particularly bRGCs and bIPCs, may underlie core features of both DS and ASD. Given the critical role these progenitors play in generating upper-layer excitatory neurons, their dysregulation could plausibly contribute to altered excitatory/inhibitory balance, a feature found in both ASD and DS. Unraveling the developmental timing and progenitor-specific vulnerabilities common to these disorders may help to identify effective therapeutic windows and strategies.

## Concluding remarks and future perspectives

Our understanding of neocortical development and circuitry has traditionally emphasized externally driven processes, often disregarding the notion that small shifts in intrinsically encoded (genetic, epigenetic and/or cellular) self-organizing principles within the germinal zones might play a significant role. Yet, incorporating deep knowledge of progenitor biology into the conceptual framework of brain function is consistent with conservative evolutionary pressures and allows for complexity to arise either incrementally or in short “bursts.”

The study of cortical neurogenesis has progressed dramatically since the initial discoveries of the principles of progenitor divisions and the later demonstration of different molecularly and morphologically defined progenitor groups. Molecular profiling and imaging tools have begun to resolve the true diversity and function of different progenitor lineages as well as the mechanisms by which NPCs contribute to circuit development and complex function.

One of the primary goals of neuroscience is to describe how the human neocortex and its developmental sequence evolved over time. Comparative neuroanatomical and developmental approaches, leveraging contemporary methodologies, are now elucidating the different progenitor lineages and their functions across lissencephalic and gyrencephalic species. Given the linkages now appreciated between a neuron’s lineage, its firing properties, and its connectivity profile, the increasing number of precursor types and/or states uncovered in primate genomics studies indicates that progenitor diversity may play a large but overlooked role in producing the complexity of brain circuits. While confirmation studies on these new additional precursor groups is still underway, the role of precursor heterogeneity in brain function is a clear and tractable path towards understanding both normal and abnormal brain development.

Much remains to be discovered about how cellular lineages diverge early in development and how this contributes both to cortical circuitry and to the specific dysfunctions seen in neurodevelopmental disorders. With specific classes of cortical progenitors now implicated in human neocortex expansion and evolution, as well as in the etiology of neurodevelopmental disorders such as DS and ASD, the future of research in neocortical development promises to uncover key details of both basic neuroscience and the mechanisms of neurological disorders. Altogether, advances in progenitor cell biology may help determine the full extent to which human neocortical development diverges from other mammals and the specific features that generate its uniqueness.
